# Marine Application Evaluation of Monocular SLAM for Underwater Robots

**DOI:** 10.3390/s22134657

**Published:** 2022-06-21

**Authors:** Yang Zhang, Li Zhou, Haisen Li, Jianjun Zhu, Weidong Du

**Affiliations:** 1 Acoustic Science and Technology Laboratory, Harbin Engineering University, Harbin 150001, China; zhyang@hrbeu.edu.cn (Y.Z.); zhujianjun@hrbeu.edu.cn (J.Z.); duweidong@hrbeu.edu.cn (W.D.); 2 Key Laboratory of Marine Information Acquisition and Security, Harbin Engineering University, Ministry of Industry and Information Technology, Harbin 150001, China; 3 College of Underwater Acoustic Engineering, Harbin Engineering University, Harbin 150001, China; 4 School of Marine Technology and Geomatics, Jiangsu Ocean University, Lianyungang 222005, China; zhoulilyg@aliyun.com

**Keywords:** visual SLAM, distortion correction, dark channel prior, image enhancement, underwater robot

## Abstract

With the development of artificial intelligence technology, visual simultaneous localization and mapping (SLAM) has become a cheap and efficient localization method for underwater robots. However, there are many problems in underwater visual SLAM, such as more serious underwater imaging distortion, more underwater noise, and unclear details. In this paper, we study these two problems and chooses the ORB-SLAM2 algorithm as the method to obtain the motion trajectory of the underwater robot. The causes of radial distortion and tangential distortion of underwater cameras are analyzed, a distortion correction model is constructed, and five distortion correction coefficients are obtained through pool experiments. Comparing the performances of contrast-limited adaptive histogram equalization (CLAHE), median filtering (MF), and dark channel prior (DCP) image enhancement methods in underwater SLAM, it is found that the DCP method has the best image effect evaluation, the largest number of oriented fast and rotated brief (ORB) feature matching, and the highest localization trajectory accuracy. The results show that the ORB-SLAM2 algorithm can effectively locate the underwater robot, and the correct distortion correction coefficient and DCP improve the stability and accuracy of the ORB-SLAM2 algorithm.

## 1. Introduction

Simultaneous localization and mapping (SLAM) is a key technology for mobile robots to achieve autonomous navigation, which has been applied and become a research hotspot in the field of underwater robots. SLAM helps underwater robots to achieve ranging, location and obstacle avoidance functions, complete the task of target location, and improve the level of intelligence. The core idea of SLAM is to achieve the pose estimation and environment map construction of autonomous underwater vehicles (AUVs) or remote-operated vehicles (ROVs) by sensor data without prior information, and to localize the underwater robot based on the environment map [1,2].

**Figure 1:**
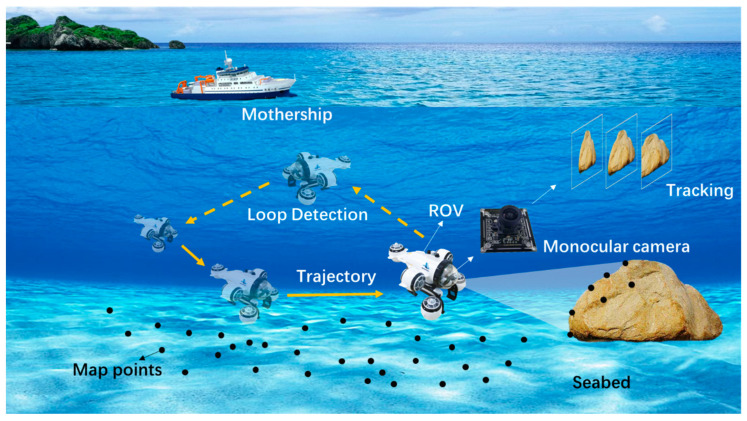
Schematic diagram of underwater visual SLAM.

SLAM uses sensor data to estimate pose, and the principles and methods vary from sensor to sensor. When SLAM first emerged, the ground environment generally used lasers and the underwater environment used sonar, which were relatively simple and straightforward ways to obtain distance [3,4]. As computer vision research advanced and processor power increased, increasingly more researchers began to use cameras as sensors [5], as shown in Figure 1. Cameras have wide compatibility, so the visual SLAM algorithm is relatively easy to develop and port. Compared with positioning methods such as ultra-short baselines and inertial navigation systems, visual SLAM has the advantages of inexpensive sensors, simple installation and operation, and environment map construction. As a pure visual positioning scheme, monocular vision is a hot trend in future research.

There are relatively mature algorithms for visual SLAM, such as ORB-SLAM [6] and LSD-SLAM [7], which have been widely used. Based on the ORB-SLAM algorithm framework, some new SLAM algorithms have been proposed, and these works aim to reduce the limitations of the algorithms and improve the map construction and localization capabilities. As far as the SLAM development stage is concerned, the algorithmic framework for SLAM problems has matured, so the research should focus on improving the practicality and stability of the algorithms and expanding the application areas, such as solving the problem of visual SLAM in underwater environments.

The underwater environment is different from land, and visual SLAM faces more challenges. In terms of seawater characteristics, seawater is a very complex mixture of chemical elements, usually containing a large amount of suspended matter and organic mixtures [8,9]. Seawater is inhomogeneous, and this inhomogeneity can lead to greater image distortion [10]. The swimming of aquatic organisms even increases the error and affects the stability. The superposition of these problems makes the conditions for normal underwater SLAM more stringent. This is the main reason why underwater visual SLAM is not yet widely used on AUVs, which face much more complex problems than on the ground.

There have been some advances in underwater visual SLAM research. In the early stage, the ROV was used to collect underwater video data, which verified the feasibility of the ORB-SLAM algorithm in the underwater environment [11]. Later, some researchers applied image processing methods to underwater SLAM, such as Retinex and histogram equalization methods [12,13]. Other researchers have compensated for attitude or visual odometry estimation errors by fusing inertial navigation or single-beam sensors, without fundamentally addressing the problem of underwater imagery.

In this paper, we improve the accuracy and stability of underwater SLAM algorithm in terms of eliminating camera imaging distortion and image enhancement and collect data from the ocean for verification. The Section 2 of this paper introduces the related theories and methods of the ORB-SLAM algorithm, image distortion correction, and image enhancement; the Section 3 is the experiment; the Section 4 is the effect evaluation; and the Section 5 is the conclusion of this paper.

## 2. Materials and Methods

### 2.1. Visual SLAM Algorithm

Many computer vision researchers have chosen to open source their algorithms, which promotes the development of visual SLAM research. Davison et al. proposed the MonoSLAM algorithm, which is the world’s first real-time monocular visual SLAM system [14]. Engle et al. proposed the LSD-SLAM algorithm, which applies the direct method to monocular SLAM with semi-dense map building. Forster et al. proposed the SVO algorithm, which is a visual odometry based on the sparse direct method [15]. Mur-Artal et al. proposed the ORB-SLAM algorithm, which divides the whole SLAM process into three threads: tracking, local mapping, and closed-loop detection, marking the gradual maturation of the visual SLAM algorithm framework.

ORB-SLAM is one of the most complete and simple algorithms, and the whole system is calculated around oriented fast and rotated brief (ORB) feature points, with features such as rotational scale invariance and fast detection. ORB-SLAM2 is upgraded from ORB-SLAM [16], supporting monocular, binocular, and RGB-D modes, and has good adaptability. The latest ORB-SLAM3 algorithm fuses inertial sensors and has no effect on this study [17]; so, this paper uses the ORB-SLAM2 algorithm. ORB-SLAM2 maintains high localization accuracy even when the robot moves and rotates rapidly. The ORB feature improves the problem that features from the accelerated segment test (FAST) detector are not directional and adopts the extremely fast binary descriptor binary robust independent elementary feature (BRIEF) to speed up the whole image feature extraction, which is a very representative real-time image feature at present [18]. ORB-SLAM uses three threads to complete SLAM, including the tracking thread for real-time tracking points, the optimization thread for local bundle adjustment, and the loopback detection and optimization thread for the global pose graph. The tracking thread extracts ORB feature points from the acquired images, compares them with the nearest keyframe, calculates the location of the feature points, and estimates the camera pose. The local bundle adjustment thread is responsible for solving more accurate camera poses and feature point spatial locations, including feature points and camera poses in local space. The global pose graph thread performs loopback detection of global maps and keyframes to eliminate cumulative errors. The loopback detection algorithm ensures that ORB-SLAM effectively eliminates cumulative errors and enables fast repositioning after tracking loss.

On the ground, the ORB-SLAM2 algorithm can achieve centimeter-level localization accuracy. The excellent characteristics of the ORB-SLAM2 algorithm are also the basis for application in underwater environments. Before the ocean experiment, we built a small pool and placed a square track to verify the applicability of the ORB-SLAM2 algorithm underwater. After calculation, the ORB-SLAM2 algorithm’s localization accuracy in this track was also at the centimeter level, which provides the basis for our ocean experiments.

### 2.2. Image Distortion Correction

**Figure 2:**
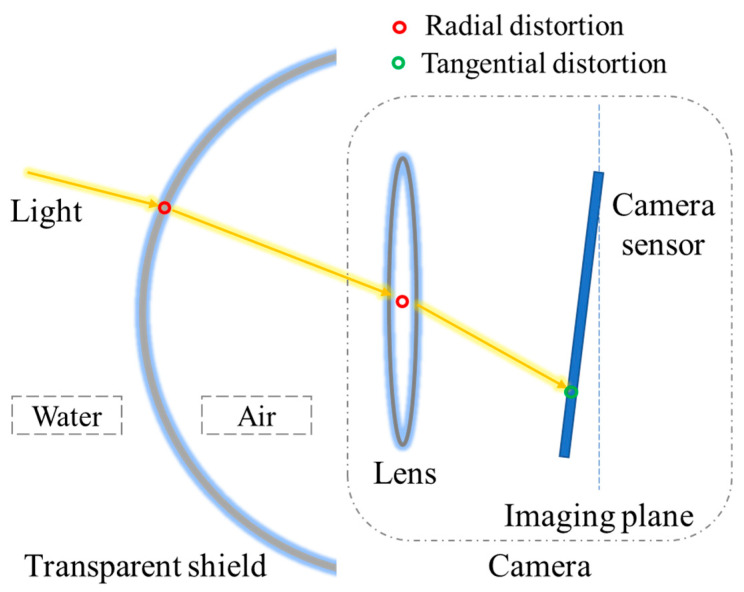
Causes of radial and tangential distortion in underwater images.

When an underwater robot uses a camera to collect images, it is affected by the robot’s own structure, camera, and environment, making the imaging distorted, and this distortion can cause errors in the SLAM accuracy [19]. The distortion comes mainly from two aspects, as shown in Figure 2. On the one hand, light propagates from one medium to another, and the phenomenon of refraction occurs. In underwater SLAM, light passes through the transparent shield of the underwater robot, the air, and the camera lens, and is finally imaged on the camera imaging plane. Propagation in different media causes a change in the direction of the light, resulting in radial distortion. On the other hand, the lens of the camera sensor itself is not mounted parallel to the light-sensitive plane, causing tangential distortion when the light passes through the lens to reach the imaging plane.

The distorted image is corrected by the distortion coefficient to obtain a distortion-free image. Whether it is radial distortion or tangential distortion, a polynomial function can be used to describe the coordinate change before and after correction [20,21]. For radial distortion, it can be corrected by quadratic and higher-order polynomial functions related to the distance from the center. Assuming that the distortion coordinate is ${\left[x,y\right]}^{T}$ and the distortion correction coordinate is ${\left[x_{\;\mathrm{corrected}\;},y_{\mathrm{corrected}}\right]}^{T}$, it can be expressed as:

(1) $$\begin{array}{l} x_{corrected}=x\left(1+k_{1}r^{2}+k_{2}r^{4}+k_{3}r^{6}\right)\\ y_{corrected}=y\left(1+k_{1}r^{2}+k_{2}r^{4}+k_{3}r^{6}\right) \end{array}$$

where *r* is the distance from the coordinate point to the origin, $k_{1}$ is the coefficient to correct the central area of the image with small distortion, $k_{2}$ is the coefficient to correct the edge area with large distortion, $k_{3}$ is the coefficient to correct the camera with large distortion such as a fisheye camera, and the $k_{1}$, $k_{2}$ coefficient can be used for ordinary cameras.

For the tangential distortion, the correction can be performed using two coefficients $p_{1}$, $p_{2}$, as follows:

(2) $$\begin{array}{l} x_{corrected}=x+2p_{1}xy+p_{2}\left(r^{2}+2x^{2}\right)\\ y_{corrected}=y+p_{1}\left(r^{2}+2y^{2}\right)+2p_{2}xy \end{array}$$

For the camera coordinate system point P(X,Y,Z), the correct position of this point in the pixel plane is obtained by combining Equations (1) and (2) with five distortion correction coefficients. The point P is projected onto the normalized image plane with coordinates ${\left[x,y\right]}^{T}$. The point is processed by radial and tangential distortion correction coefficients to obtain the correct coordinates on the image, which can be expressed as:

(3) $$\begin{array}{c} x_{corrected}=x\left(1+k_{1}r^{2}+k_{2}r^{4}+k_{3}r^{6}\right)+2p_{1}xy+p_{2}\left(r^{2}+2x^{2}\right)\\ y_{corrected}=y\left(1+k_{1}r^{2}+k_{2}r^{4}+k_{3}r^{6}\right)+p_{1}\left(r^{2}+2y^{2}\right)+2p_{2}xy \end{array}.$$

The corrected point is projected to the pixel plane through the internal parameter matrix, and the correct coordinates on the image are obtained as follows:

(4) $$\begin{array}{l} u=f_{x}x_{\;corrected\;}+c_{x}\\ v=f_{y}y_{corrected}+c_{y} \end{array}.$$

Image distortion correction can calculate the distortion coefficient through camera calibration, but the distortion coefficient in water and air is not the same, so it needs to be calibrated in a similar underwater environment.

### 2.3. Underwater Image Enhancement Algorithm

The purpose of underwater image enhancement is to meet the underwater SLAM requirements, using image enhancement algorithms to reduce image noise and increase contrast. It was observed that underwater images have a great similarity to haze images, especially with turbid water or poorly lit seafloor. Therefore, the algorithm of dark channel prior (DCP) is chosen to study in this paper, and the performance of three methods, including contrast-limited adaptive histogram equalization (CLAHE), median filtering (MF), and DCP, in underwater SLAM are compared [22,23,24].

In the field of computer vision, there is a broad model to describe haze images, as shown in the following equation:

(5) I(x)=J(x)t(x)+A(1−t(x)).

where I(x) and J(x) represent the haze and haze-free images, respectively; t(x) refers to the transmittance; and A is the global atmospheric light value. It can be seen from the model that the dehazing algorithm is a process of obtaining A and t(x) by means of other methods to solve J(x) by knowing the haze image I(x).

The DCP algorithm uses as prior knowledge that there is always a channel with a lower gray value among the RGB channels of image pixels according to the statistical properties of the faze image. The expression for the dark channel is:

(6) $$J^{dark}(x)=\underset{c\in \{R,G,B\}}{\min}\left\{\underset{y\in \Omega (x)}{\min}(J^{c}(y))\right\}.$$

where $J^{dark}$ denotes the dark channel and $J^{c}$ refers to the RGB channel of the recovered image J. According to the prior knowledge of dark channel dehazing, it is obtained that $J^{dark}$ converges to 0.

The transmittance can be estimated from $I^{c}(y)$ and $A^{c}$. He et al. introduced a constant θ, usually taking the value 0.95, keeping a small amount of haze in the distant image, and the transmittance expression is:

(7) $$\tilde{t}(x)=1-\theta \underset{c}{\min}\left\{\underset{y\in \Omega (x)}{\min}(I^{c}(y)/A^{c})\right\}.$$

In the DCP algorithm, the atmospheric light value *A* is solved based on the dark channel map, and the top 0.1% of the pixel points with the highest luminance in the map are obtained. Among the first 0.1%-pixel points, the pixel points with the highest luminance in the haze image I(x) are found and its value is used as the atmospheric light value. Then, the transmittance t(x), the atmospheric light value A, and the haze image I(x) are all known quantities, and the haze-free image J(x) is obtained; the expression is:

(8) $$J(x)=((I(x)-A)/\max\{t(x),t_{0}\})+A.$$

Among them, in order to prevent the transmittance t(x) from approaching 0, $t_{0}$ is the lower limit of transmittance and is taken as 0.1. The traditional method of finding transmittance tends to lead to the white edge effect in haze-free images, and He proposed a soft matting method to refine the transmittance. The soft matting method is time-consuming, and later, guided filtering was proposed to refine the transmittance, which is more practical.

## 3. Experiment

The purpose of the experiment is twofold: the first is to obtain the distortion correction coefficient of the camera, and the second is to collect image data for SLAM. In this paper, we use an ROV-mounted monocular camera for image acquisition, the acquisition and storage process are more efficient and convenient, and the SLAM approach for offline dataset is repeatable.

### 3.1. Obtain the Camera Distortion Correction Coefficients

The distortion correction coefficients were obtained using the camera calibration experiment, which was performed in the pool due to the limitation of the test conditions. The calibration experiment was performed with the same ROV as the sea test, the model White Shark Mini, with the parameters shown in Table 1. The White Shark Mini is a consumer-grade ROV, with a monocular camera mounted in a glass enclosure directly in front of the ROV, and a fill-in light on the left and right sides. It can be manipulated to complete forward, backward, up, down, and turning movements, and its small size makes it easy to operate and carry.

**Figure 3:**
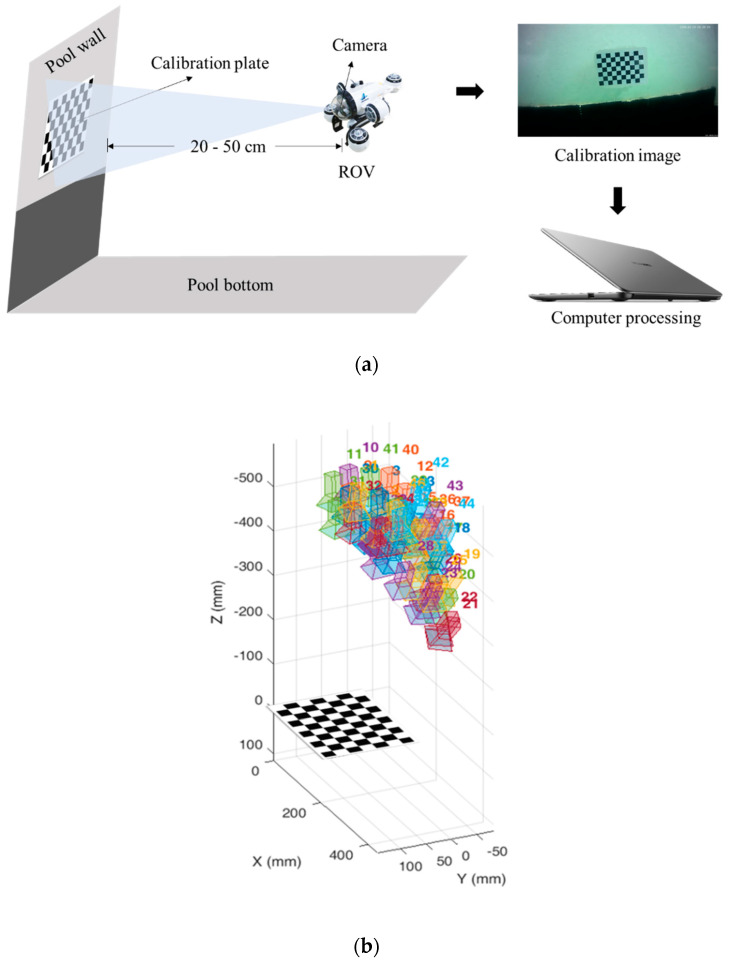
Camera calibration. (**a**) Schematic diagram of the process of acquiring calibration images by the ROV in the pool; (**b**) The position of the camera relative to the calibration plate during image acquisition.

In this paper, a single-plane checkerboard grid calibration method was used to obtain the distortion correction coefficients and camera internal parameters. The calibration plate is a 10 × 7 grid, and the ROV was manipulated to take 49 images in the pool. The image shooting positions are shown in Figure 3. More than 3 images are required for normal calibration, and the more images, the more accurate the calibration results.

The 49 images were solved, in which only 2 images with large reprojection errors were removed, and the camera internal reference K and the distortion correction coefficients $k_{1},k_{2},p_{1},p_{2},k_{3}$ were obtained, as follows:

(9) $$K=\left[\begin{array}{ccc} f_{x} & 0 & c_{x}\\ 0 & f_{y} & c_{y}\\ 0 & 0 & 1 \end{array}\right]=\left[\begin{array}{ccc} 775.270656 & 0.000000 & 293.603808\\ 0.000000 & 775.814093 & 295.499837\\ 0.000000 & 0.000000 & 1.000000 \end{array}\right],$$

(10) $$\left[k_{1},k_{2},p_{1},p_{2},k_{3}\right]=\left[-0.446910,0.298707,-0.002197,0.001014,0.000000\right],$$

where $f_{x}$ and $f_{y}$ denote the number of pixels represented by *f* in the imaging plane with a focal length *f*; $c_{x}$ and $c_{y}$ denote the pixels that offset the origin of the physical imaging plane. Since the monocular camera is not a fisheye camera, the $k_{3}$ result is 0.

We also calculated the camera’s internal parameters and distortion correction coefficients in the air, which are:

(11) $$K=\left[\begin{array}{ccc} f_{x} & 0 & c_{x}\\ 0 & f_{y} & c_{y}\\ 0 & 0 & 1 \end{array}\right]=\left[\begin{array}{ccc} 585.649935 & 0.000000 & 311.878140\\ 0.000000 & 584.794956 & 312.834346\\ 0.000000 & 0.000000 & 1.000000 \end{array}\right],$$

(12) $$\left[k_{1},k_{2},p_{1},p_{2},k_{3}\right]=\left[-0.455836,0.239824,0.004961,0.000828,0.000000\right].$$

Obviously, the results in air and water are not consistent, which is why the underwater camera intrinsic and distortion correction coefficients were obtained. The results in water were used in the ORB-SLAM2 algorithm.

### 3.2. Data Collection and Preprocessing

The ORB-SLAM2 algorithm can run in real time and handle offline datasets, and the results are consistent in both ways. For the convenience of data processing, SLAM is performed in the form of offline datasets. The White Shark Mini ROV was used to collect video data in the experiment, which was located in the waters of Okinawa, Japan.

**Figure 4:**
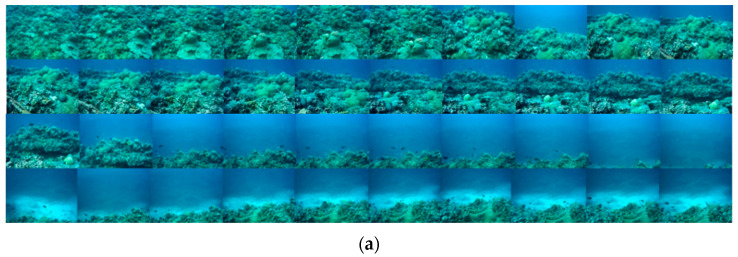

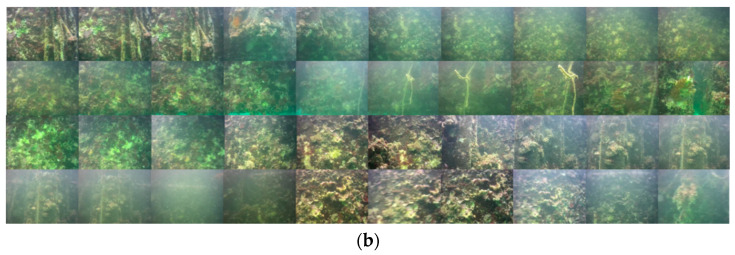
Partial results of decomposition of video data into images. (**a**) Partial results of Test 1 data decomposition; (**b**) Partial results of Test 2 data decomposition.

Not all the data collected by ROV can be initialized in the ORB-SLAM2 algorithm. In order to compare different cases, two video data with different image quality were selected, which were collected at two locations with different water qualities. Among them, the water quality of the data collected on the coral reefs in the shallow sea is relatively clear, and the data set is named Test 1; the water quality of the data collected near the wharf is turbid, and the data set is named Test 2. Firstly, the experimental data was preprocessed; the ORB-SLAM2 algorithm cannot process the video data directly, and the video was decomposed into images by the frame rate. The results are shown in Figure 4 and Table 2. Since the frame rate is 25 frames per second, the number of decomposed images is not strictly equal to the product of time and frame rate, and the number is normal in the range of ±13 frames.

Judging from the image characteristics of the two datasets, the Test 1 dataset images are clearer, and the coral reefs on the seabed are clearly visible, so the need for image enhancement is smaller in the actual SLAM. The Test 2 dataset images have turbid water quality, which can be regarded as blurred and haze images, and are more suitable for comparing the effects of different image enhancement methods.

## 4. Effect Evaluation

### 4.1. Image Enhancement Effect Evaluation

**Figure 5:**
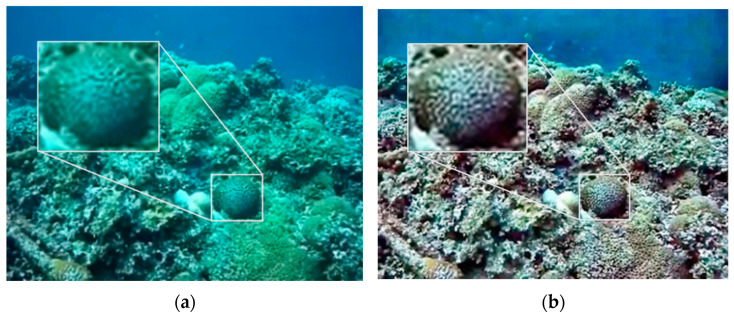

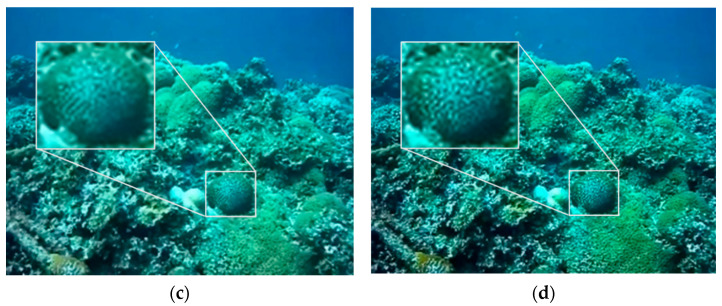
Test 1 image enhancement results. (**a**) Original image; (**b**) CLAHE; (**c**) MF; (**d**) DCP.

**Figure 6:**
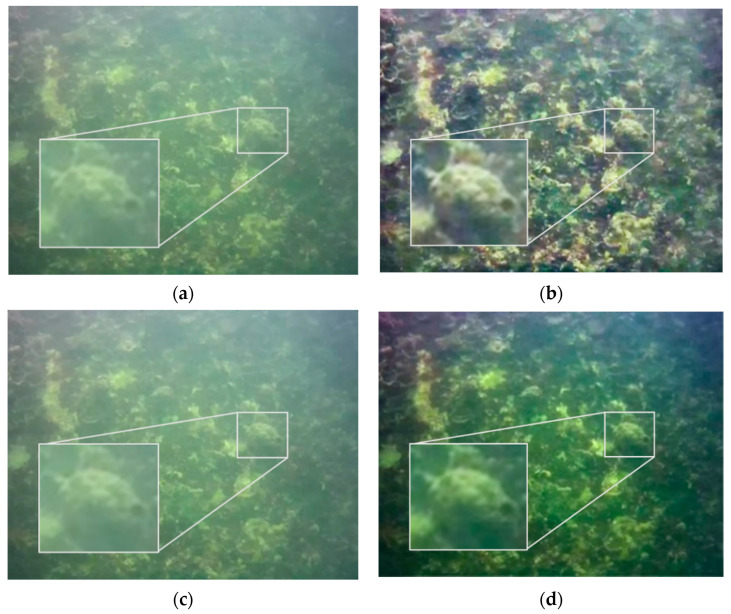
Test 2 image enhancement results. (**a**) Original image; (**b**) CLAHE; (**c**) MF; (**d**) DCP.

One image in each of the two datasets was selected and processed by CLAHE, MF, and DCP, respectively, and the processing results are shown in Figure 5 and Figure 6. In order to compare the effect of image enhancement, this paper adopted three methods for evaluation, namely subjective evaluation, the PSNR and SSIM index, and ORB feature matching.

Subjective evaluation draws conclusions by observing and analyzing images. It can be seen from the results that the contrast of the two groups of CLAHE results has improved, and the texture is clearer, but there is a large chromatic aberration compared with the original image while new noise has been introduced. The MF algorithm characteristics make the processed image blurrier than the original image, but, overall, the MF result is closer to the original image. The DCP results are the best. In Test 1, the local texture is richer, and the image as a whole becomes clearer. In Test 2, the haze feeling of the image is largely reduced, the image is clearer, and the details are more prominent.

To further objectively evaluate the image enhancement effect, two traditional image evaluation metrics, the peak signal-to-noise ratio (PSNR) and structural similarity (SSIM), were used. PSNR is a widely used image evaluation criterion that depends on the error between the corresponding pixels of the original image and the enhanced image. The larger PSNR indicates less image distortion, and PSNR can be expressed as:

(13) $$PSNR=10\log_{10}({\left(2^{n}-1\right)}^{2}/MSE),$$

where *n* is the number of bits of pixels, and generally, the grayscale image *n* is taken as 8. MSE denotes the mean square error between the original image and the enhanced image.

SSIM evaluates the similarity of an image in terms of brightness, contrast, and structure, and takes values in the range [0, 1]. The larger the SSIM value, the smaller the distortion. SSIM can be expressed as:

(14) SSIM=L(I,J)×C(I,J)×S(I,J),

Among them, L(I,J) represents brightness, C(I,J) represents contrast, and S(I,J) represents structure.

After calculation, the evaluation index results of the three methods of CLAHE, MF, and DCP are shown in Table 3.

As can be seen from Table 3, in the Test 1 dataset, the DCP method obtained the highest PSNR and SSIM, which are 26.808 and 0.980, respectively. It shows that when the image is relatively clear, the image distortion obtained by the DCP method is the smallest. The CLAHE method has the largest distortion. In the Test 2 dataset, the MF method obtained the highest PSNR and SSIM: 44.911 and 0.986, respectively. It shows that when the image is blurred, the MF method is the closest to the original image. Compared with the original image, DCP has the largest distortion, but this does not mean that the processed image becomes blurred: it is the performance of the successful processing of the blurred image.

**Figure 7:**
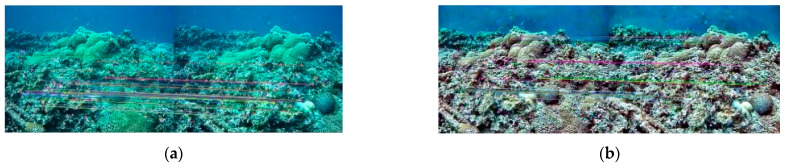

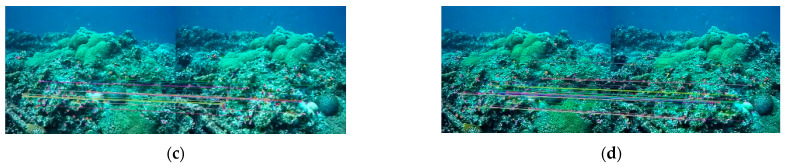
Feature matching results of the Test 1 dataset. (**a**) Original image; (**b**) CLAHE; (**c**) MF; (**d**) DCP.

**Figure 8:**
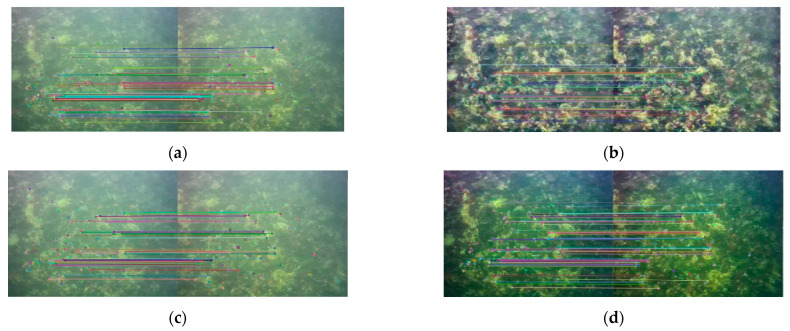
Feature matching results of the Test 2 dataset. (**a**) Original image; (**b**) CLAHE; (**c**) MF; (**d**) DCP.

The ORB-SLAM2 algorithm extracts ORB feature point matching in the image for pose estimation, so it is obviously beneficial to complete SLAM if more ORB feature points can be matched. In the two test datasets, two adjacent images were found for feature point matching, the number of feature matches between the two frames of images was calculated, and the changes after image enhancement were compared. Two frames of images were found in the Test 1 and Test 2 datasets, respectively, with slight pose changes between them. The ORB feature point extraction and matching results are shown in Figure 7 and Figure 8.

**Figure 9:**
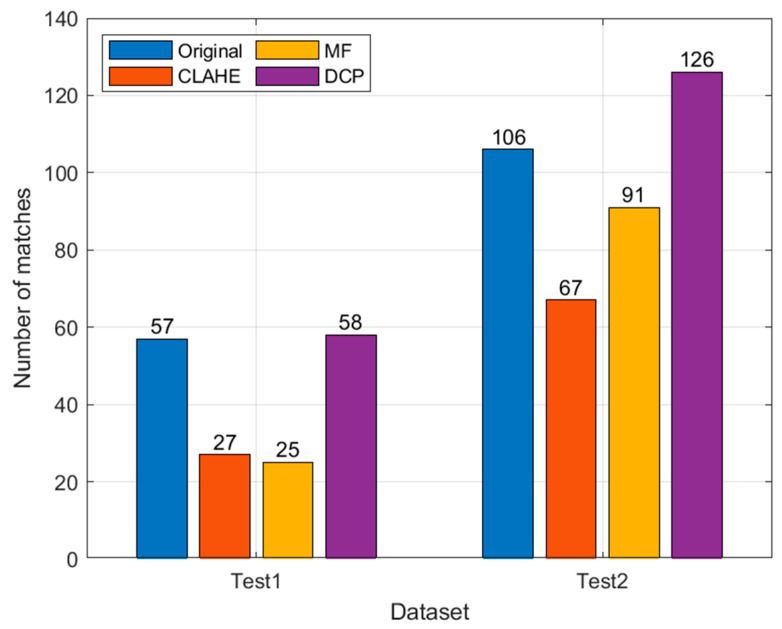
Number of feature matches.

The number of image feature matches is shown in Figure 9.

**Figure 10:**
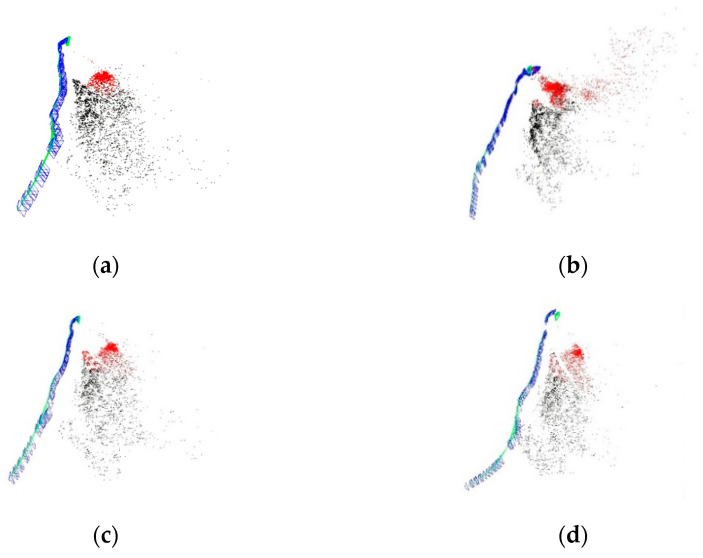
SLAM results for the Test 1 dataset. (**a**) Original; (**b**) CLAHE; (**c**) MF; (**d**) DCP.

As can be seen from the results in Figure 10, the CLAHE and MF methods do not improve the number of image features matched but are lower than the original image. For MF, compared to the original image, the image edges were smoothed and the contours were more blurred, so the number of feature matches was less, and the effect of this change was mitigated in Test 2, where the image itself was more blurred. The number of feature matches of the CLAHE method instead decreased when the image contrast increased, probably due to its introduction of new noise. The DCP method has the largest number of ORB feature matching, especially in the Test 2 dataset: the DCP has 20, 59, and 35 more features than the original image, CLAHE, and MF, respectively, and the feature matching effect is the best.

The image of Test 1 is clearer than Test 2, but the number of ORB feature matches is less than that of Test 2 because the image texture of Test 2 is richer than that of Test 1. In Figure 7, about ¼ of the image is transparent seawater, and the lack of texture makes it difficult to extract feature points, which also leads to fewer feature matches than Figure 8. The two datasets in this paper have different scenes and different numbers of images, so there is no way to compare them directly. We chose to compare the differences between the image enhancement and the original image results, thus assessing the effectiveness of the image enhancement method in clear (Test 1) and turbid (Test 2) seawater.

### 4.2. SLAM Effect Evaluation

**Figure 11:**
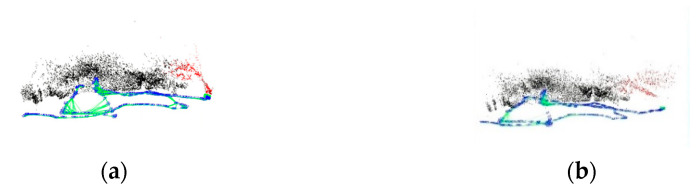

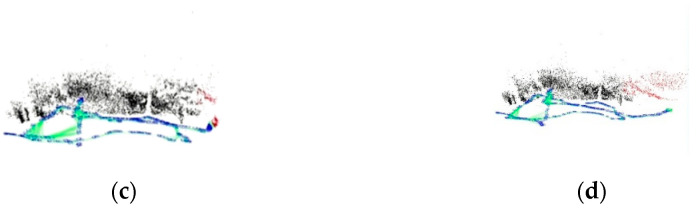
SLAM results for the Test 2 dataset. (**a**) Original; (**b**) CLAHE; (**c**) MF; (**d**) DCP.

To further evaluate the effect of image enhancement in underwater SLAM, images from the Test 1 and Test 2 datasets were imported into the ORB-SLAM2 algorithm to obtain the trajectory and point cloud map of the ROV. Among the ORB-SLAM2 algorithm results, there are mainly camera poses and trajectories, and sparse point cloud maps. Blue represents the camera pose, green the camera motion trajectory, black the global point cloud map, and red the local point cloud map. The point cloud map and the trajectory of the camera constitute the relative positioning, which is the main basis for ROV positioning. The ORB-SLAM2 results are shown in Figure 10 and Figure 11.

**Figure 12:**
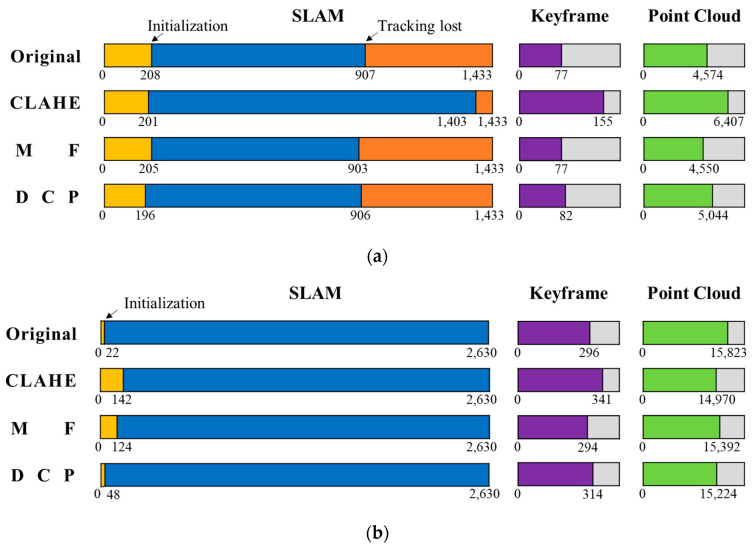
SLAM running result data. (**a**) Test 1 dataset; (**b**) Test 2 dataset.

The result graph shows that after image enhancement, the key frame trajectories differ from the original data, but the overall trend is still consistent. Throughout the SLAM process, the main parameters of initialization, tracking lost, key frames, and point clouds are counted, as shown in Figure 12. In the Test 1 dataset, the SLAM initialization times are all around the 200th frame, and the initialization time difference is less than 1 s based on the 25 frames per second of the camera. The DCP initialization time is the fastest, with the initialization completed at frame 196. The original data, MF, and DCP tracking is lost around frame 900, with a similar number of key frames and point clouds. CLAHE suffers from tracking loss at frame 1403, the longest SLAM duration, and the highest number of key frames and point clouds. No relocation occurred after four tracking losses. In the Test 2 dataset, the original data and DCP were the fastest to complete initialization, at frames 22 and 48, respectively, much faster than the CLAHE and MF methods, and there was no tracking lost. The CLAHE obtained the most keyframes and the least point clouds.

Initialization is the beginning of SLAM, and faster initialization means more image feature points are matched and the image quality is better. In both datasets, DCP has the shortest initialization time among the three image enhancement methods. The tracking loss is due to the fact that not enough feature points were extracted, and the system started to wait to enter relocation. In the Test 1 dataset, where tracking loss occurs, the CLAHE method performs best. In ORB-SLAM2, the keyframe selection rules are to determine whether the distance from the previous keyframe is more than 20 images, whether the current frame tracking is less than 50 point clouds, and whether the current frame tracking is less than 90% of the reference keyframe point cloud. CLAHE has the most keyframes, especially in Test 1, which is almost twice as many as the other results. The point clouds are determined by the number of feature points of keyframes, and the number of point clouds varies due to different keyframe selection and keyframe feature points, but the overall quantitative difference is not significant.

**Figure 13:**
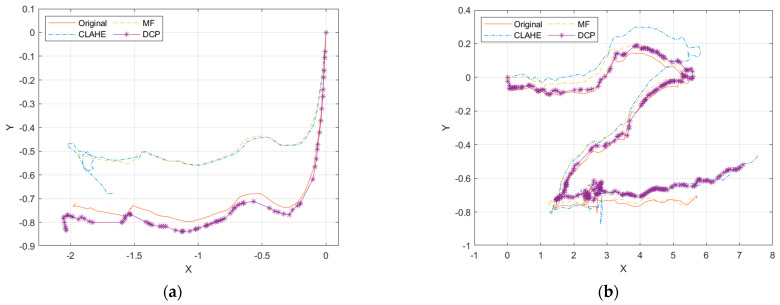
Keyframe motion trajectory. (**a**) Test 1; (**b**) Test 2.

There is a scale uncertainty problem in monocular vision, and it is impossible to judge whether the distance is meters or centimeters, but the initialized fixed scale can still be used as a basis for judging the accuracy. The X and Y axes of the keyframe trajectory were extracted, and the results are shown in Figure 13.

**Figure 14:**
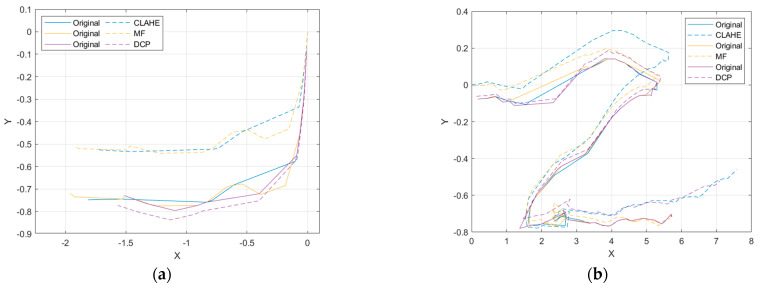
Comparison of the same keyframe trajectory with the original data. (**a**) Test 1; (**b**) Test 2.

It can be visualized from the figure that the Test 1 trajectory results diverge, with the CLAHE and MF trajectories shifting shortly after SLAM initialization, resulting in higher overlap of the original and DCP trajectories and higher overlap of the CLAHE and MF trajectories. In Test 2, the CLAHE, MF, and original deviate more in the first half, and the CLAHE, DCP, and original deviate more in the second half. After image enhancement, the SLAM keyframe sequence numbers are not consistent, so the same keyframes need to be selected to calculate the root mean square error (RMSE) of the image enhancement trajectory and the original trajectory. The keyframes of the image enhancement and the original data are marked with the same color, as shown in Figure 14.

In the Test 1 dataset, the RMSEs of CLAHE, MF, and DCP are: 0.175, 0.167, and 0.030, respectively, and the error between the DCP and original trajectory is the smallest. In the Test 2 dataset, the RMSEs of CLAHE, MF, and DCP are: 0.280, 0.131, and 0.196, respectively, and the error between the MF and original trajectory is the smallest. It is not that DCP did not perform well in Test 2, because in the first half of the trajectory, the RMSEs of CLAHE, MF, and DC are: 0.166, 0.122, and 0.060, respectively, and DCP still has the lowest RMSE. Since the Test 2 dataset itself is more ambiguous, the accuracy of the motion trajectory in DCP is higher.

From the SLAM evaluation results, it can be seen that the localization accuracy of DCP is higher than that of CLAHE and MF, and the stability is also better in the SLAM algorithm. Combined with the results of the image enhancement evaluation, DCP had the best overall performance. In clear waters, DCP maintains image clarity and SLAM positioning accuracy, improving the image texture details. In turbid waters, DCP improves the image clarity, the number of ORB feature matches, and the SLAM localization accuracy. Therefore, DCP improves the application conditions of visual SLAM, and visual SLAM can be applied in sea with worse water quality.

## 5. Conclusions

Visual SLAM is a relatively cheap option for underwater robot localization, but the harsh underwater environment leads to poor accuracy and stability of SLAM. The purpose of this study was to address the current problems of underwater vision SLAM in practical applications, propose corresponding solutions, and conduct experimental validation to provide a direct and reliable reference for underwater vision SLAM applications. The first measure was to calibrate the camera in the underwater environment to obtain distortion correction coefficients to ensure the accuracy of the positional estimation. In the pool environment, the ROV captured 49 images continuously, and obtained the accurate internal parameters of the monocular camera and five distortion correction coefficients. The second measure was image enhancement processing, which improves the clarity and contrast of underwater blurred images and maintains the stability and accuracy of the SLAM system. Three methods, CLAHE, MF, and DCP, were selected for comparison, and two datasets collected by ROV in Okinawa waters were processed separately. Compared with CLAHE and MF, DCP showed the best image evaluation index, the largest number of feature point matching, and the smallest positioning trajectory error in the ORB-SLAM2 algorithm, the most accurate positioning results, and better stability. This paper shows that the ORB-SLAM2 algorithm can be effectively used as a localization method for underwater robots, and after image distortion correction and DCP image enhancement, it offers the possibility of practical application of visual SLAM on underwater robots. In the future, we will improve the calculation of the algorithm speed, integrate the SLAM algorithm and image enhancement into the ROV platform, and achieve real-time localization and map construction. At the same time, the inertial navigation system will be equipped on the ROV to compare the accuracy difference between the visual SLAM and inertial navigation system, and further evaluate the localization performance of visual SLAM underwater.

## Figures and Tables

**Table 1:** ROV and monocular camera parameters table.

	Item	Parameter
ROV	Size	416 × 355 × 210 mm
	Working depth	75 m
	Weight in air	2.8 kg
	Speed	3 kn
Camera	Type	Monocular
	Resolution	1920 × 1080
	Focal length	3.6 mm
	PTZ angle	±55°

**Table 2:** Details of the two datasets.

Dataset	Duration	Frame Rate	Number of Images	Image Quality
Test 1	57 s	25 fps	1433	High
Test 2	105 s	25 fps	2630	Low

**Table 3:** PSNR and SSIM calculated values for the CLAHE, MF, and DCP methods.

Methods	Test 1		Test 2	
	PSNR	SSIM	PSNR	SSIM
CLAHE	21.596	0.879	21.179	0.891
MF	25.596	0.911	**44.911**	**0.986**
DCP	**26.808**	**0.980**	16.759	0.769

## Data Availability

Not applicable.
